# LncRNA Chaer Prevents Cardiomyocyte Apoptosis From Acute Myocardial Infarction Through AMPK Activation

**DOI:** 10.3389/fphar.2021.649398

**Published:** 2021-07-15

**Authors:** Zhiyu He, Xiaojun Zeng, Deke Zhou, Peiying Liu, Dunzheng Han, Lingling Xu, Tong Bu, Jinping Wang, Mengmeng Ke, Xiudi Pan, Yipeng Du, Hao Xue, Dongfeng Lu, Bihui Luo

**Affiliations:** ^1^ Department of Cardiology, The First Affiliated Hospital of Guangzhou Medical University, Guangzhou, China; ^2^ Traditional Chinese Medicine Hospital of Gaozhou, Department of Cardiology, Gaozhou, China

**Keywords:** lncRNA, myocardial infarction, apoptosis, AMPK, cardioprotecion

## Abstract

Long non-coding RNA (lncRNA) is widely reported to be involved in cardiac (patho)physiology. Acute myocardial infarction, in which cardiomyocyte apoptosis plays an important role, is a life-threatening disease. Here, we report the lncRNA Chaer that is anti-apoptotic in cardiomyocytes during Acute myocardial infarction. Importantly, lncRNA Chaer is significantly downregulated in both oxygen-glucose deprivation (oxygen-glucose deprivation)-treated cardiomyocytes *in vitro* and AMI heart. *In vitro*, overexpression of lncRNA Chaer with adeno virus reduces cardiomyocyte apoptosis induced by OGD-treated while silencing of lncRNA Chaer increases cardiomyocyte apoptosis instead. *In vivo*, forced expression of lncRNA Chaer with AAV9 attenuates cardiac apoptosis, reduces infarction area and improves mice heart function in AMI. Interestingly, overexpression of lncRNA Chaer promotes the phosphorylation of AMPK, and AMPK inhibitor Compound C reverses the overexpression of lncRNA Chaer effect of reducing cardiomyocyte apoptosis under OGD-treatment. In summary, we identify the novel ability of lncRNA Chaer in regulating cardiomyocyte apoptosis by promoting phosphorylation of AMPK in AMI.

## Introduction

Cardiovascular disease, especially ischemic heart disease, is the leading cause of morbidity and mortality around the world (McAloon et al., 2016). And elevating incidence of cardiovascular disorders brings a huge burden to thousands of families (Heusch and Gersh, 2017). The increasing cardiomyocyte apoptosis has a considerable contribution to cardiomyocyte death at the early stage of AMI, thus triggers cardiac injury (Matsui et al., 2010) (Teringova and Tousek, 2017). Furthermore, cardiomyocyte apoptosis takes part in cardiac remodeling and heart failure process after myocardial infarction (Orogo and Gustafsson, 2013). Hence, it is worthy to inhibit the cardiomyocytes apoptosis against AMI-induced cardiac injury. However, the regulating mechanism of apoptosis that contributes to the cardiac injury in AMI remains to be elucidated. AMP-activated protein kinase (AMPK), consisting of α, β, γ subunits, is considered as an important regulator in underlying mechanisms of myocardial infarction (Gu et al., 2018). *Sun et al.* had proved AMPK a protective molecule in myocardial infarction by reducing cardiac apoptosis and interstitial fibrosis (Sun et al., 2013). Loss of AMPK may accelerate ischemia/reperfusion injury while its activation can protect heart again ischemia (Zhang et al., 2017) (Duan et al., 2017). Targeting AMPK may be a promising treatment in ameliorating cardiac ischemia injury.

Long noncoding RNA (lncRNA), made up of more than 200 nucleotides, can directly bind to DNA for the recruitment of epigenetic modulators and act as a decoy for transcription factors. Thus, this in turn modulates splicing or interact with proteins fir achieving specific functions (Wang and Chang, 2011; Moran et al., 2012). In addition, some lncRNAs serve as sponges for microRNAs to regulate specific process (Paraskevopoulou and Hatzigeorgiou, 2016). Recently, lncRNA has been proved as a powerful epigenetic regulator in cardiovascular diseases (Poller et al., 2018; Sallam et al., 2018). Researchers have investigated the roles of lncRNA in regulating cardiomyocyte apoptosis after AMI to reduce cardiac injury and improve cardiac functions. *Li X et al.* reported lncRNA Mirt1 attenuated AMI injury by reducing cardiomyocytes apoptosis and inflammatory cell infiltration through inhibition of the NF-κB cell signaling pathway (Li et al., 2017). Another lncRNA, named ZFAS1, was found to be involved in the modulation of cardiomyocyte apoptosis in AMI through a ZFAS1-miR-150-C-reactive protein axis (Wu et al., 2017). These studies suggest that lncRNA is a potential therapeutic target to inhibit cardiomyocytes apoptosis thus attenuate cardiac injury in AMI.

LncRNA Chaer was reported to accelerate the process of cardiac hypertrophy. LncRNA Chaer combined with PRC2 promotes the transcription of pro-hypertrophy genes which should have been inhibited by the PRC2 complex at basal condition (Wang et al., 2016). Interestingly, researchers have identified that the activation of intrinsic-mediated caspase is highly linked with cardiomyocytes hypertrophy. The intrinsic apoptotic pathway can be inhibited at several points and can serve a leading role in the prevention of cardiomyocytes hypertrophy during agonist stimulations which form a close relationship between apoptosis and hypertrophy (Putinski et al., 2013). Studies have suggested that there are multiple parallel and staggered signaling pathways between hypertrophy and apoptosis (Fortuño et al., 2003; Vanempel and De Windt, 2004). The hypertrophy-related signal pathways affect cardiomyocytes apoptosis to a certain extent (Shiraishi et al., 2004; Vanempel and De Windt, 2004; Pillai et al., 2014; Ikeda and Sadoshima, 2016). According to the reported data, JAK/STAT, and calcineurin/NFAT pathways exert a pro‐survival regulatory effect and also have a link in cardiac hypertrophy (Manukyan et al., 2010). So, it is reasonable to believe that hypertrophy-related lncRNA Chaer might have a significant contribution in modulating cardiomyocytes apoptosis. As we mentioned above, cardiomyocytes apoptosis is an important process at the early stage of AMI, it is of great interest that whether hypertrophy-related lncRNA Chaer is involved in this process. Here, we investigate whether hypertrophy-related lncRNA Chaer takes part in the modulation of cardiomyocytes apoptosis at the early stage of AMI and the mechanism behind.

## Methods and Materials

### NMCMs

#### Isolation, Cell Culture

Neonatal mice cardiac myocytes (NMCMs) were taken from 1 to 3 days old mice pups (C57BL/6) (Li et al., 2017). The hearts of pups were adequately minced and were digested enzymatically, using trypsin (0.25%) and collagenase I (0.15%, Gibco). The cell suspension was centrifuged, followed by suspending in DMEM, supplemented with FBS (10%). Then the suspension (cells) was added into each Petri plate. The plates were incubated for 90 min to attach the fibroblasts to the bottom of the plates. Non-adherent NMCMs were separated and seeded in another Petri plate, followed by two days of incubation in a humidified incubator (37°C with 95% air and 5% CO_2_). For the oxygen-glucose deprivation (OGD) treatment, cells were seeded in serum-free medium. The seeded cells were then kept in an incubator, set at 37°C, 5% CO_2_, 1% O_2_, and 94% N_2_ for 6, 12, and 24 h.

### Adenovirus and Small Interfering RNA Transfection

The adenovirus overexpression Chaer was synthesized by Genechem (Shanghai, China). Isolated NMCMs were seeded at 90% confluence and cultured for 48 h. For adenovirus transfection, adenovirus-Chaer (Adv-Chaer) or adenovirus-negative control (Adv-NC) was treated with the cells with a multiplicity of infections (50 MOI). Small interfering RNA (siRNA) for silencing Chaer (siRNA-Chaer) and the corresponding negative controls (siRNA-NC) were designed and constructed by GenePharma (Shanghai China) (Li et al., 2019). The siRNA sequences targeting the sequence of Chaer transcript were as follows: 5’-GAG​CCA​AAA​ACC​AA-CAA​GGA-3’. Lipofectamine^®^ RNAiMAX reagent (Invitrogen, Carlsbad, United States) was employed for NMCMs transfection, as suggested by the manufacturer’s protocol.

### Acute Myocardial Infarction Models

8 to 10 weeks-old mice (C57BL/6) were provided by Guangzhou University of Chinese Medicine. The underline *in-vivo* experiments on mice were carried out following the Guidelines for the Care and Use of Laboratory Animals, and its approval was provided by the Animal Ethics Review Committee of Guangzhou Medical University Hospital. Mice were randomly divided into a sham-operated group, a MI group, an AAV9-NC group and an AAV9-Chaer group (n = 7–11). AMI models were evoked via permanent ligation of the left anterior descending coronary artery (LADCA) (Wu et al., 2017). Firstly, isoflurane was used to anesthetize the mice and was then placed on a heating pad (37°C) to keep the mice warm. To expose the heart, the thoracic cavity was open at the third costal space, while the LADCA branches were ligated at the inferior edge of the left atrial auricle (2 mm) with an 8–0 suture needle. Sham-operated mice underwent the same protocol with no ligation. After AMI for 24 h, the infarct border zones of myocardial tissues were excised to evaluate qRT-PCR and immunoblotting experiments.

### Injection of AAV9 Into Adult Mice

Adenoviral-associated vector 9 (AAV9), overexpression lncRNA Chaer was synthesized by Genechem (Shanghai, China) (Prasad et al., 2011). Five-week-old mice were anesthetized with isoflurane. Then, 20 μl containing 3.15×10^9^ viral genomes (vg) of AAV9 negative controls (AAV9-NC) (n = 7–11) or AAV9 overexpression Chaer (AAV9-Chaer) (n = 7–11) were randomly injected into each mouse from the external jugular vein. At 4 weeks post-injection, a model of acute myocardial infarction was established in mice.

### Examination of Cardiomyocytes Apoptosis

The TUNEL assay was used for the evaluation of apoptotic cells in cultured cells as well as tissue sections, as suggested by the procedure, established by the manufacturer (*In Situ* Cell Death Detection Kit, TMR red, Sigma-Aldrich, United States). Confocal microscopy (Nikon Eclipse Ni-u, Japan) was employed for the visualization of apoptotic and non-apoptotic cardiac myocytes. For every sample, the nuclear density was identified through the counting of nuclei (stained with DAPI) in five various fields. The apoptotic myocytes were counted and then results were identified by dividing the total number of TUNEL-positive cells by the total number of DAPI-positive cells.

### CCK-8 Assay

In a 96-well plate, the suspension (100μL/well) of cells was added and incubated at 37°C for 24 h. This was followed by addition of 10 μL CCK8 (Glpbio, United States) to each well and further incubated for 1–4 h. The absorption and reference wavelengths were 450 nm and 600–650 nm respectively. Each sample was tested in triplicate.

### Echocardiography

One day post-MI and before surgery, a Vevo 770 Imaging System of Visual Sonics (Toronto, Canada) was employed with a 30-MHz linear array transducer for the evaluation of two-dimensional and M-mode echocardiograms of the mice (n = 10–11). The percent ejection fraction (EF) and fractional shortening (FS) were measured using M-mode PSAX (parasternal short axis) view while left ventricular anterior wall; diastolic (LVAWd), left ventricular anterior wall; systolic (LVAWs), left ventricular posterior wall; diastolic (LVPWd), left ventricular posterior wall; systolic (LVPWs) were measured via PLAX (parasternal long axis).

### Measurement of Myocardial Infarct Size

24-h post-AMI, 200 μL Evans Blue (1%) was administered through the external jugular vein and then after 5 min, the hearts were rapidly taken and were kept at −40°C and then were cut into slightly thicker sections (2 mm) transversely, followed by incubation at 37°C for 40 min in 1-percent 2, 3, 5-triphenyltetrazoliumchloride (TTC, Sigma-Aldrich, United States) (Rong et al., 2020) (n = 7). The infarct area (INF, pale white) and the area at risk (AAR, brick red) were evaluated digitally via ImageJ software (Media Cybernetics, United States). The ratio of INF to AAR and AAR to LV was then recorded.

### Quantitative Real-Time PCR

TRIzol Reagent (Invitrogen, Carlsbad, United States) was used for the isolation of total RNA from NMCMs, followed by the cDNA construction with the help of Reverse Transcription Kit (Takara, Shanghai, China), as suggested by the manufacturer’s protocol. To evaluate the lncRNAs and mRNAs expressions, qRT-PCR experiments were conducted via SYBR Green PCR Kit (Takara, Shanghai, China). The underlined primer sequences were used for lncRNA Chaer: forward: 5′-TCC AAT GAG GGA AGC GAA GC-3′, reverse 5′-GTC CGA TGC CAG TTC CAG TT′; 18S: forward 5′-GGT GCA TGG CCG TTC TTA-3′, reverse 5′-TGC CAG AGT CTC GTT CGT TA-3′. The relative expression level was determined via the 2^−△△Ct^ method.

### Western Blot Analysis

RIPA buffer along with phosphatase and PMSF were used for the digestion of Cardiac cells and tissues for 0.5-h, followed by centrifugation. Total protein quantification and separation were carried out via SDS-PAGE (12%). Then proteins were moved onto a PVDF-membrane, followed by blockage with skimmed milk (5%). The proteins were treated with primary antibodies (at 4°C) along with the CST, United States products including β-Tubulin (1:2,000), Cleaved caspase 3 (1:500), caspase 3 (1:1,000), AMPK (1:1,000) and p-AMPK (1:1,000). After overnight incubation, membranes were incubated with secondary antibodies for 2 h. Blots were visualized by Chemiluminescence kit (Invitrogen, Carlsbad, United States) and analyzed by ImageJ software (Media Cybernetics, United States).

### Statistical Analysis

Graphpad Prism-6 and SPSS 16.0 statistical package (SPSS, Inc., Chicago, IL, United States) were employed for the evaluation of statistical data. The obtained data were indicated as mean ± SEM (standard error mean). All assays were repeated (at least 3 times). The one-way analysis of ANOVA was employed to compare several groups and then a student’s t-test was conducted to identify the *p*-value between two groups. A *p*-value of less than 0.05 was found to be considerable.

## Results

### Downregulation of LncRNA Chaer in the Infarct Border Zone of AMI Induced Mice and the Cardiomyocytes Under OGD Treatment

**FIGURE 1:**
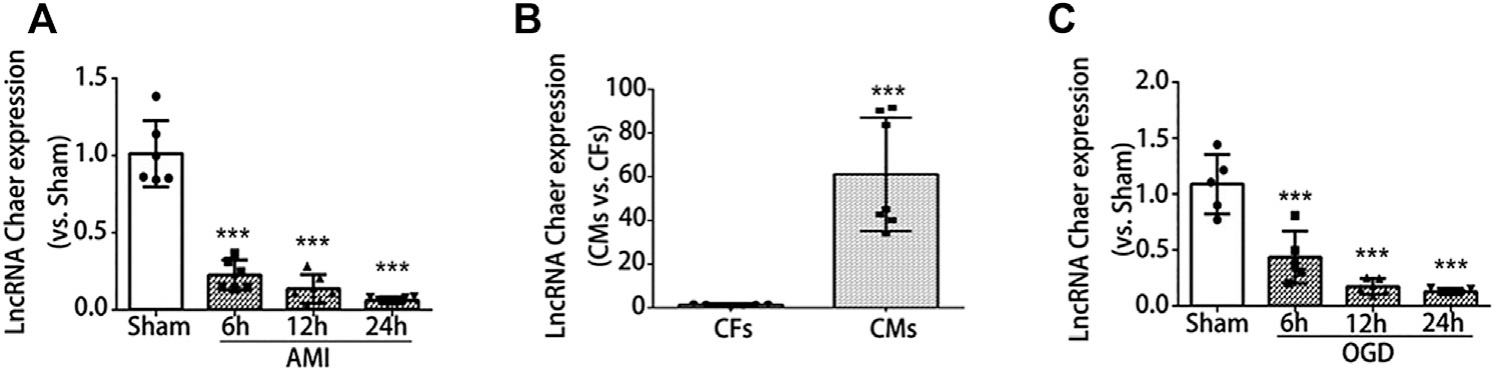
LncRNA Chaer expression was downregulated both in the infarct border zone after AMI and in the cardiomyocytes under OGD treatments. **(A)** mice were subjected to AMI, and sacrificed after 6, 12 and 24 h, then qRT-PCR was conducted to test the relative levels of lncRNA Chaer in the border zone of mouse heart. ***p < 0.001 vs. sham, n = 6. **(B)** Expression of lncRNA Chaer in primary cultured cardiomyocytes and fibroblasts. ***p < 0.001 CMs vs. CFs, n = 6. **(C)** Primary cultured CMs were treated with OGD for 6, 12, 24 h, then qRT-PCR was performed to value the expression of lncRNA Chaer in each group. ***p < 0.001 vs. sham, n = 5.

For the investigation of lncRNA Chaer expression in AMI induced mice, first, we developed the AMI model in mice. Evans blue and TTC staining results showed that the myocardial infarct zone was found to be clear at 24 h after AMI surgery (Supplementary Figure S1A). The heart undergoes an inflammatory phase and can be distinguished with enhanced cardiomyocyte death, including apoptosis in the first few days after AMI (Liehn et al., 2011). A comparatively elevated level of apoptotic process was observed in the border zones (Wang et al., 2018). Similarly, the expression of cleaved caspase 3 was considerably upregulated in the infarct border zones (Supplementary Figure S1B). It's meaningful to inhibit the apoptosis in the border zones to save the heart function. Then we tried to figure out the relative potential regulator. Here, we observed the expression of lncRNA Chaer was significantly reduced and obtained its lowest levels at 24 h post-AMI injury in the infarct border zones (Figure 1A) which revealed that lncRNA Chaer has a possible role in the regulation of apoptosis in border zones.

To further determine the expression of lncRNA Chaer in specific cell types, qRT-PCR was conducted for samples derived from primary cultured cardiomyocytes and fibroblasts. Results indicated that the expression of lncRNA Chaer in cardiomyocytes was more than 60 folds higher as compared to cardiac fibroblasts, suggesting a potential role of lncRNA Chaer in cardiomyocytes (Figure 1B). In this study, we used an oxygen-glucose deprivation (OGD) model of cardiomyocytes to study ischemic cell death under hypoxic conditions and simulate the conditions of myocardial ischemia *in-vivo*. Immunoblot analysis demonstrated that the expression of cleaved caspase 3 was considerably upregulated in cardiomyocytes treated with OGD (Supplementary Figure S1D). Meanwhile, the expression of lncRNA Chaer decreased significantly and reached the lowest level at 24 h in cardiomyocytes treated with OGD (Figure 1C). These data showed differential expression of lncRNA Chaer after OGD treatment or AMI surgery and imply that lncRNA Chaer may play a role in the related process of cardiomyocyte death.

### Downregulation of lncRNA Chaer Increases Apoptosis in Cardiomyocytes Treated With OGD

**FIGURE 2:**
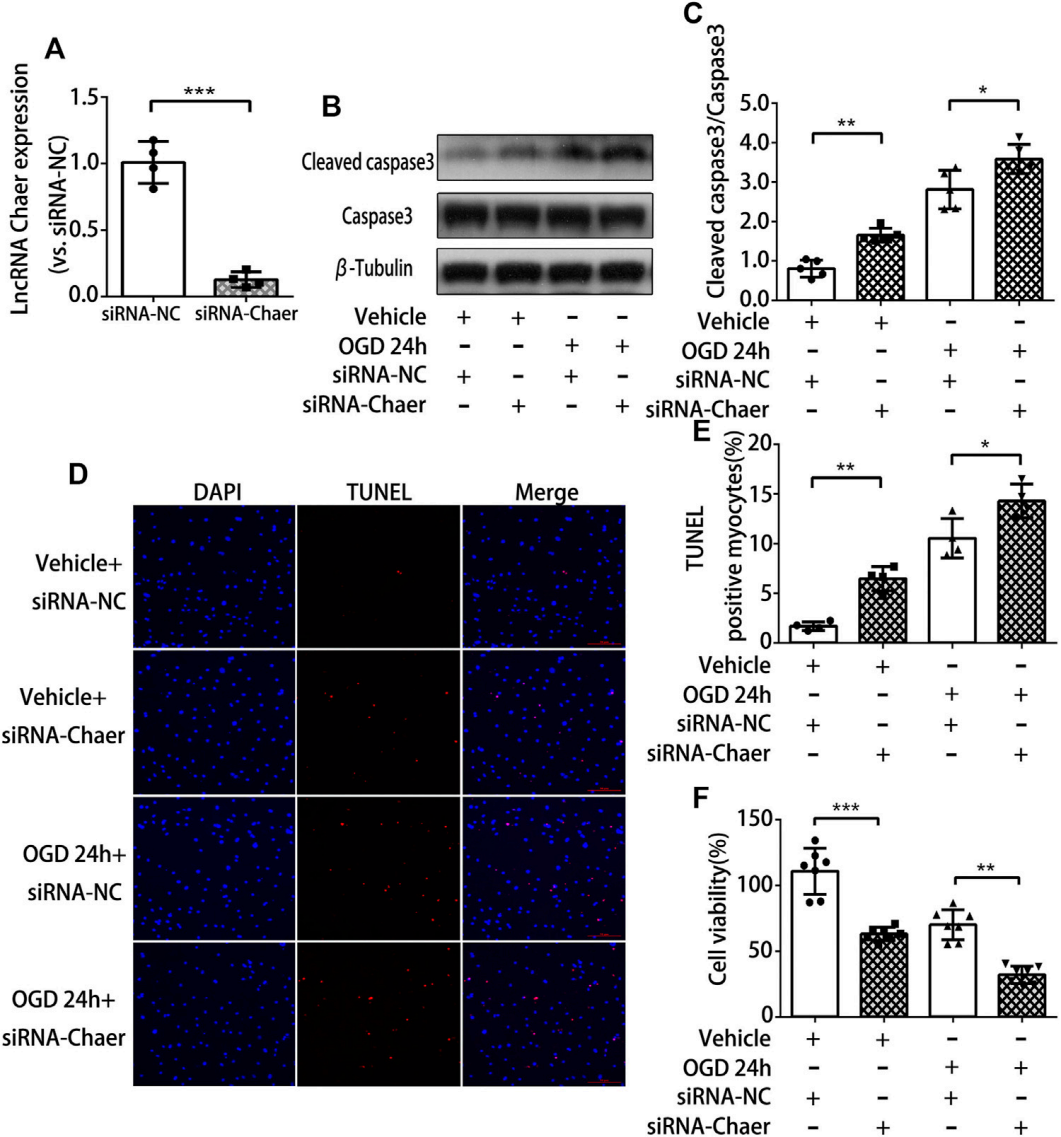
Downregulation of lncRNA Chaer increases apoptosis in cardiomyocytes treated with OGD. **(A)** Knockdown of lncRNA Chaer with SiRNA Chaer in cardiomyocytes. ***p < 0.001 vs. siRNA-NC, n = 4. **(B, C)** After SiRNA transfection, CMs were treated with OGD for 24 h, then western blotting was applied to test the expression of apoptosis-related protein caspase 3 and cleaved caspase 3 in cardiomyocytes. *p < 0.05, **p < 0.01, n = 5. **(D, E)** Representative TUNEL-stained images of siRNA-Chaer-infected cardiomyocytes after treatments of OGD. Red, TUNEL-positive nuclei; blue, DAPI; scale bar = 50 μm. Quantification of TUNEL-positive cardiomyocytes. *p < 0.05, **p < 0.01, n = 4. **(F)** Effect of siRNA-Chaer on cell viability after 24 h OGD treatments by CCK8 analysis. **p < 0.01, ***p < 0.001, n = 7.

To examine the function of lncRNA Chaer in cardiomyocytes treated with OGD, siRNA was used to knock down the expression of lncRNA Chaer in cardiomyocytes. Transfection of siRNA-Chaer was found to be successfully decreased the expression of lncRNA Chaer in cardiomyocytes, compared to the negative control (NC) group (Figure 2A). And we found that the downregulation of lncRNA Chaer significantly augmented the expression of cleaved caspase 3 (Figures 2B,C) and the BAX/Bcl-2 ratio (Supplementary Figures S2A,B) under OGD treatments vs. the NC group. Correspondingly, the TUNEL assay confirmed that siRNA-Chaer groups were more vulnerable to cardiomyocytes apoptosis under OGD treatments than that in the NC group (Figures 2D,E). Furthermore, CCK-8 assay demonstrated that knockdown of lncRNA Chaer aggravated OGD-induced cardiomyocytes injury (Figure 2F). These results indicated that the downregulation of lncRNA Chaer could increase OGD-induced cardiomyocytes apoptosis.

### Overexpression of lncRNA Chaer Decreases Apoptosis in Cardiomyocytes, Treated With OGD

**FIGURE 3:**
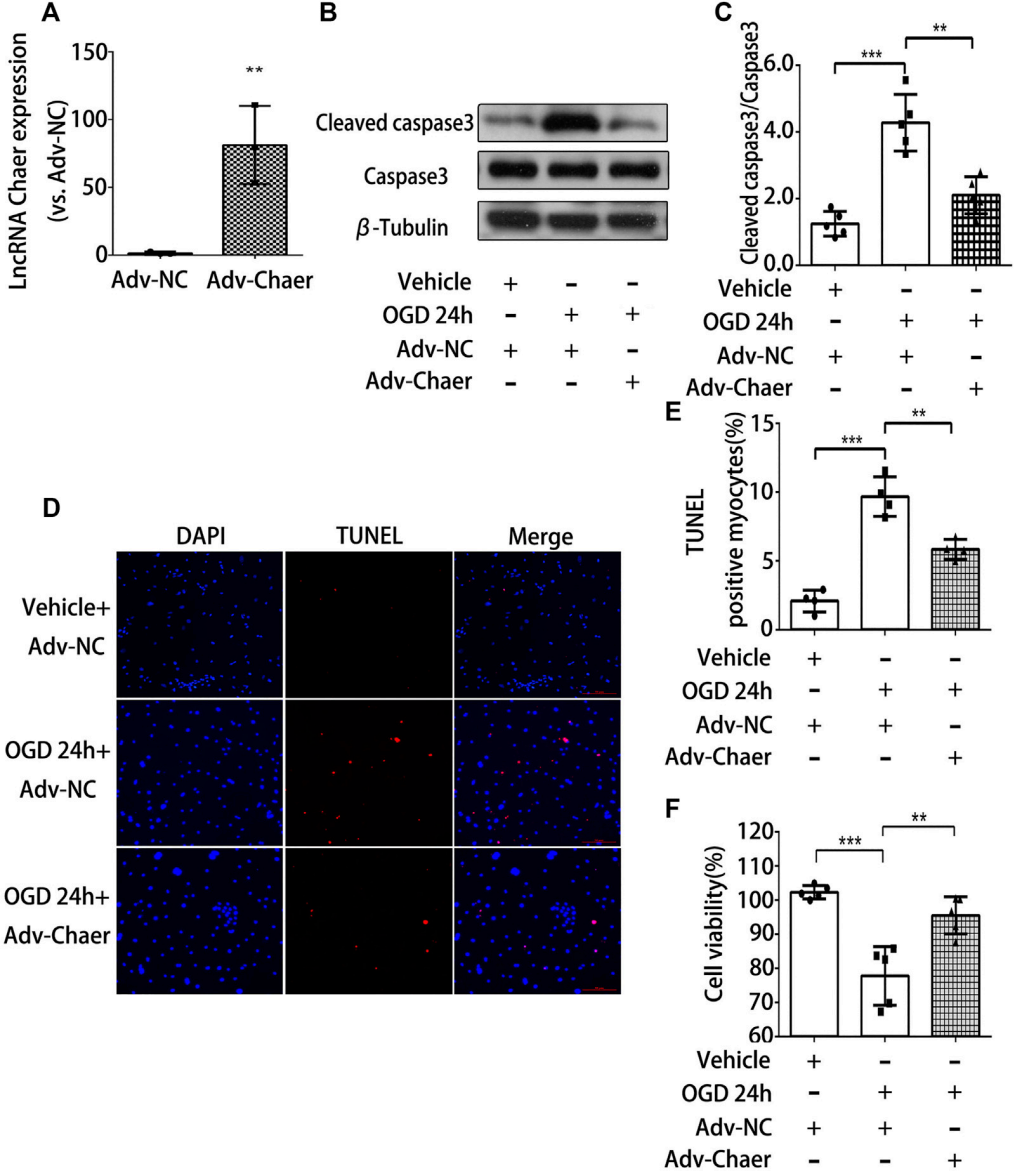
Overexpression of lncRNA Chaer decreases apoptosis in cardiomyocytes treated with OGD. **(A)** Elevated expression of lncRNA Chaer by Adv-Chaer in cardiomyocytes. **p < 0.01 vs. Adv-NC, n = 3. **(B, C)** After Adv-Chaer/Adv-NC transfection, CMs were treated with OGD for 24 h, then Western blotting was performed to test the expression of Cleaved caspase3 expression in cardiomyocytes. **p < 0.01, ***p < 0.001, n = 5. **(D, E)** Representative TUNEL-stained images of Adv-Chaer-infected cardiomyocytes after treatments of OGD. Red, TUNEL-positive nuclei; blue, DAPI; scale bar = 50 μm. Quantification of TUNEL-positive cardiomyocytes. **p < 0.01, ***p < 0.001, n = 4. **(F)** Effect of Adv-Chaer on cell viability after 24h OGD treatments by CCK8 analysis. **p < 0.01, ***p < 0.001, n = 5.

To further validate the role of lncRNA Chaer in cardiomyocytes treated with OGD, we transfected the cardiomyocytes with adenoviral vectors encoding lncRNA Chaer (Adv-Chaer). Quantitative real-time PCR was conducted to validate that the lncRNA Chaer expression was effectively upregulated with 50 MOI Adv-Chaer (Figure 3A). LncRNA Chaer elevated expression considerably halted cleaved caspase 3 stimulation (Figures 3B,C) and the BAX/Bcl-2 ratio (Supplementary Figures S2C,D) under OGD treatments. Additionally, the TUNEL assay showed that overexpression of lncRNA Chaer exhibited more resistance to cardiomyocytes apoptosis under OGD treatments (Figures 3D,E). Moreover, the high expression of lncRNA Chaer elevated the viability of cardiomyocytes under OGD treatments (Figure 3F). It is clear from the results that overexpression of lncRNA Chaer could block OGD‐induced cardiomyocytes apoptosis. Taken together, lncRNA Chaer could effectively modulate cardiomyocytes apoptosis in response to OGD conditions.

### Overexpression of lncRNA Chaer Inhibits Cardiomyocytes Apoptosis From Acute Myocardial Infarction in Mice

**FIGURE 4:**
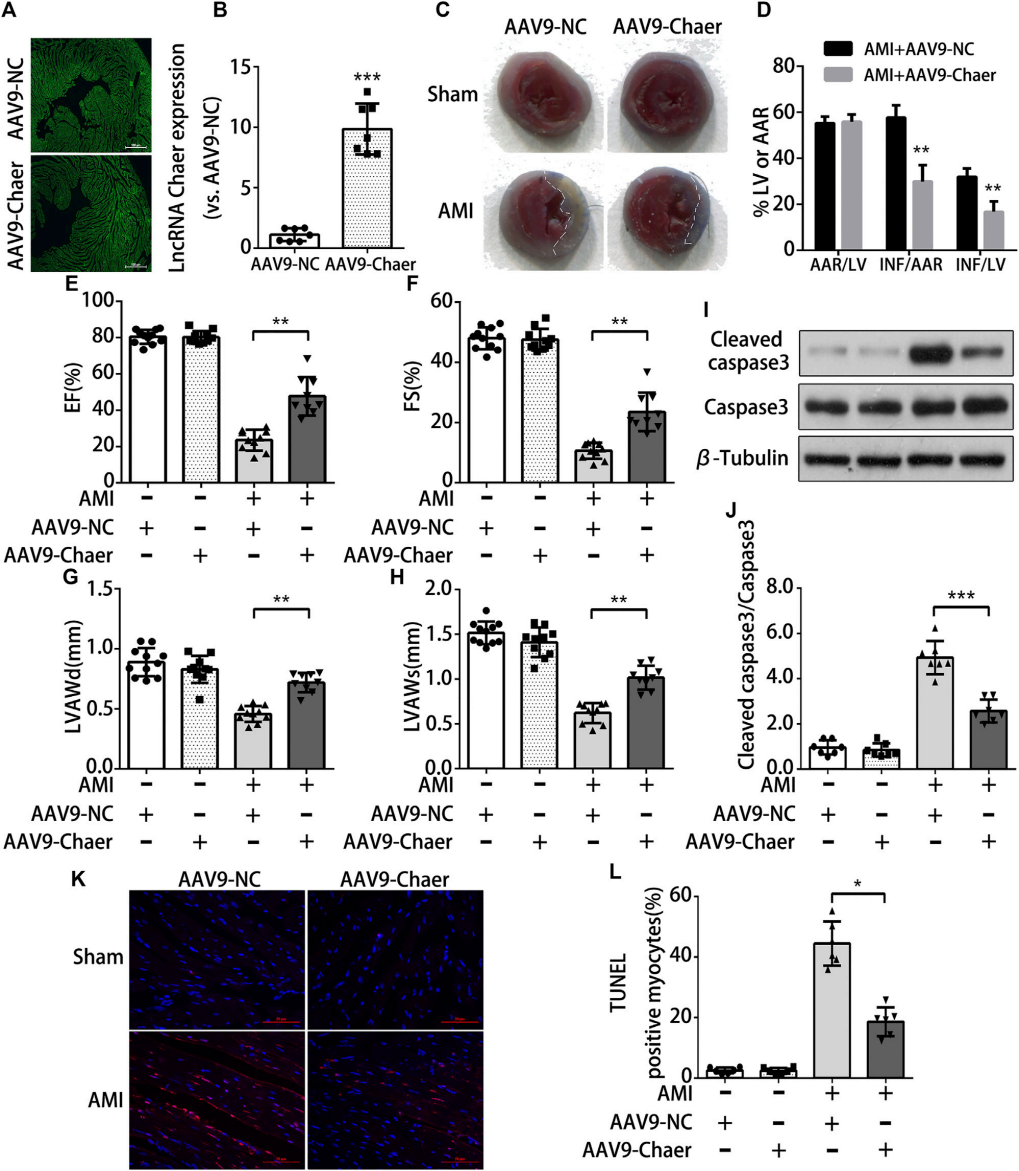
Overexpression of lncRNA Chaer protects cardiomyocytes apoptosis from acute myocardial infarction in mice **(A)**AAV9-mediated LncRNA Chaer was injected into the external jugular vein before LAD coronary ligation. After 4 weeks, the hearts were harvested for GFP fluorescence measurement to confirm the successful AAV9 transfection. **(B)** qRT-PCR analysis of lncRNA Chaer expression in mouse cardiac tissue. ***p < 0.001 vs. AAV9-NC, n = 7. **(C, D)** Representative images of middle cross sections of cardiac Evans blue/TTC staining. Blue color represents the non-infarct area, red indicates the area at risk and white indicates the infarct area. Quantification of infarct area. AAR, Area at risk; INF, Infarct size; LV, Left ventricle; **p < 0.01, n = 7. **(E–H)** Functional and quantitative echocardiographic analysis of the left ventricle in mice. The EF%, FS%, LVAWd and LVAWs are shown, **p < 0.01, n = 10–11. **(I, J)** Representative Western blotting and quantification of Cleaved caspase3 expression in myocardial tissues of each group. ***p < 0.001, n = 7. **(K, L)** TUNEL staining of cell apoptosis in myocardial tissues of each group. Red, TUNEL-positive nuclei; blue, DAPI; scale bar = 50 μm. Quantification of TUNEL-positive cardiomyocytes. *p < 0.05, n = 6.

To further investigate whether lncRNA Chaer has cardioprotective effects *in-vivo*, the method of adenovirus-associated vector 9 (AAV9)-Chaer-mediated gene delivery was adopted. This method been reported to be effective in providing cardiac genetic intervention. Through injecting AAV9 into the external jugular vein of mice for four weeks, 6 μm cryosections were prepared and evaluation of eGFP expression via fluorescence microscopy suggested that AAV9 efficiently transduced into cardiomyocytes (Figure 4A). In parallel, lncRNA Chaer was significantly upregulated in ventricular myocardium (Figure 4B). Next, the size of myocardial infarction in mouse cardiac tissue was identified by Evans blue and TTC dual staining (Figure 4C). Overexpression of lncRNA Chaer did not make a significant difference in the cardiac remodeling among sham-operated mice. However, the area of myocardial infarction was found to be decreased in AMI mice following AAV9-Chaer treatment, as compared with the AMI mice upon AAV9-NC treatment (Figure 4D). Afterward, cardiac function was evaluated via echocardiography in mice after AMI injury. The percentage of EF and FS of left ventricular was reduced after AMI injury, while it was increased in AAV9-Chaer mice in comparison with that of AAV9-NC mice (Figures 4E,F). Furthermore, the left ventricular diastolic anterior wall (LVAWd) and left ventricular systolic anterior wall (LVAWs) were significantly reduced in AMI mice, compared with the sham-operated mice. Importantly, overexpression of lncRNA Chaer enlarged LVAWd and LVAWs in comparison with the AAV9-NC treatment group (Figures 4G,H). There was no significant difference in the left ventricular diastolic posterior wall (LVPWd) and left ventricular systolic posterior wall (LVPWs).

Subsequently, the obtained data from immunoblot analysis revealed an elevation in cleaved caspase 3 expression in the AMI mice in comparison with the mice with the sham operation. The AMI mice upon AAV9-Chaer treatment were found to be decreased in the expression of cleaved caspase 3 when compared to AMI mice upon AAV9-NC treatment (Figures 4I,J). Furthermore, TUNEL staining indicated that the apoptosis rate of cardiomyocytes in the AMI mice upon AAV9-Chaer treatment presented a decline in correlation with the AMI mice upon AAV9-NC treatment (Figures 4K,L).

In short, overexpression of lncRNA Chaer, serving as a cardioprotective regulator, could inhibit cardiomyocytes apoptosis from acute myocardial infarction and reduce infarction area, thus improved the heart function in AMI mice.

### LncRNA Chaer Attenuates Cardiomyocytes Apoptosis Through Its Ability to Promote Activation of AMPK

**FIGURE 5:**
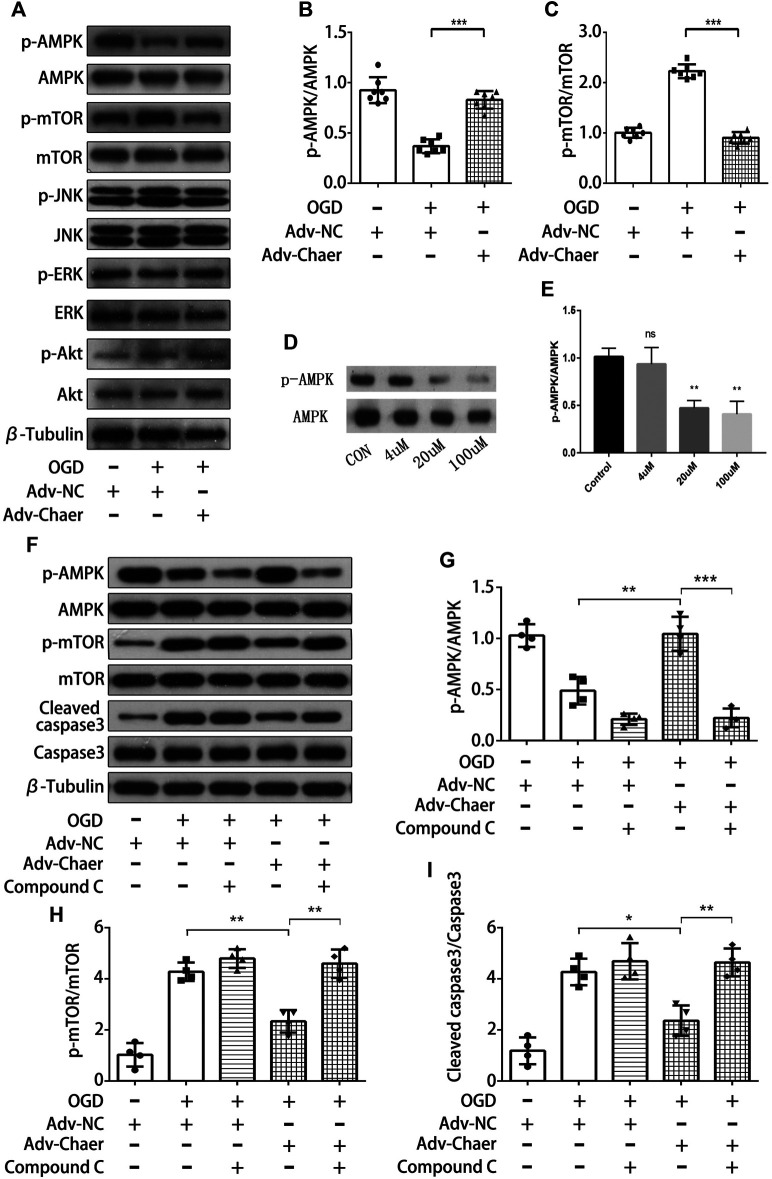
LncRNA Chaer attenuates cardiomyocytes apoptosis through its ability to promote activation of AMPK **(A)**LncRNA Chaer-stimulated phosphorylation signaling in cardiomyocytes. Changes in the phosphorylation levels of AMPK (p-AMPK), mTOR (p-mTOR), JNK(p-JNK), ERK(p-ERK) and Akt (p-Akt) under OGD treatments with overexpression of lncRNA Chaer were determined by Western blot analysis. **(B, C)** Quantitative data of AMPK (p-AMPK) and mTOR (p-mTOR) under OGD treatments with overexpression of lncRNA Chaer. ***p < 0.001, n = 7. **(D, E)** Representative Western blotting results and quantitative data of AMPK (*p*-AMPK) after Compound C (0,4,20,100 μM) treatment. **p < 0.01, n = 3. **(F–I)** Representative Western blotting and quantification of AMPK (p-AMPK), mTOR (p-mTOR) and Cleaved caspase3 expression in cardiomyocytes with Compound C and OGD treatments. *p < 0.05, **p < 0.01, ***p < 0.001, n = 4.

To investigate the potential pathways regulated by lncRNA Chaer in AMI‐induced cardiomyocytes apoptosis, we evaluated the phosphorylation of various molecules linked to apoptotic process with the overexpression of lncRNA Chaer in cardiomyocytes. Overexpression of lncRNA Chaer in cardiomyocytes stimulated the phosphorylation of AMPK (AMP-activated protein kinase) and inactivated its downstream target mTOR. In contrast, overexpression of lncRNA Chaer did not affect the phosphorylation of ERK (extracellular signal-regulated kinase), JNK (Jun N-terminal kinase), and Akt in cardiomyocytes (Figures 5A–C). To evaluate whether AMPK is involved in the antiapoptotic properties of lncRNA Chaer, cardiomyocytes were treated with compound-C, an AMPK inhibitor commonly used to suppress the AMPK pathway. As expected, AMPK phosphorylation in cardiomyocytes treated with Compound C was inhibited in a dose-dependent manner (Figures 5D,E). Overexpression of lncRNA Chaer in cardiomyocytes under OGD treatments significantly increased the levels of AMPK phosphorylation and eliminated the stimulating effects on mTOR, while treatment with Compound C reversed the inhibitory effects of lncRNA Chaer on OGD-induced cardiomyocytes apoptosis (Figures 5F–I). Therefore, lncRNA Chaer could attenuate cardiomyocytes apoptosis, at least in part, though its ability to promote the phosphorylation of AMPK.

## Discussion

LncRNA Chaer was originally discovered in the study of cardiac hypertrophy (Wang et al., 2016), which inhibited the functions of PRC2 by binding with the PRC2 complex, interfered with the targeting of the PRC2 genome, thereby regulated the transcription of its neighboring hypertrophic genes to promote cardiac hypertrophy. In pathophysiology, the balance between cell death and survival is a strictly controlled process (Vanempel and De Windt, 2004), especially in terminally differentiated cells, such as cardiomyocytes. Studies have suggested that there are multiple parallel and staggered signaling pathways between hypertrophy and apoptosis (Fortuño et al., 2003). The signaling cascades linked with hypertrophy regulates the balance between cardiomyocytes' survival and death. There is a balance between survival and hypertrophic signals on one hand, while apoptotic signals are on the other hand, and a persistent shift in this regulation results in the elevation of the apoptotic process (Shiraishi et al., 2004; Manukyan et al., 2010; Pillai et al., 2014; Ikeda and Sadoshima, 2016). Studies have suggested that stimulation of the survival pathway transduced by the IL6 receptor IL6st to the transcription factor STAT3 is important for the survival of cardiomyocytes in the face of acute oxidative stress (Sharma et al., 2012). Therefore, it is reasonable to believe that hypertrophy-related lncRNA Chaer may play a role in modulations of cardiomyocytes apoptosis. In current study, we revealed the pivotal role of lncRNA Chaer in acute myocardial infarction. First, we found that lncRNA Chaer was downregulated in both the tissues of myocardial infarction and cardiomyocytes treated with OGD. LncRNA Chaer modulated cardiomyocytes apoptosis and cell viability. Overexpression of lncRNA Chaer augmented cardiomyocytes viability after OGD treatment, whereas knockdown of lncRNA Chaer aggravated OGD-induced cardiomyocytes injury. In addition, we found that overexpression of lncRNA Chaer reduced the cardiomyocytes apoptosis and infarct area and enhanced cardiac functions in mice after AMI. Previous studies reported that lncRNA Chaer promoted cell proliferation and suppressed apoptosis, while down-regulation of lncRNA Chaer facilitated cell death under pathological stress. Therefore, lncRNA Chaer was suggested to be a factor that mediated cell fate, which is consistent with our discovery that lncRNA Chaer regulated cardiomyocytes apoptosis.

When cardiomyocytes are irreversibly injured, they are eliminated from the heart via activating intracellular apoptosis (Teringova and Tousek, 2017). This apoptotic process can lead to cellular shrinkage, chromatin condensation, the formation of apoptotic bodies, and DNA fragmentation. The elevated level of myocardial apoptosis results in loss of contractile units, which is highly associated with cardiac remodeling and cardiac dysfunction (Orogo and Gustafsson, 2013; Del Re et al., 2019). AMP-activated protein kinase (AMPK) is an important regulator of cellular metabolism and has an important contribution in myocardial apoptosis in ischemic heart disease (Qi and Young, 2015). AMPK, a serine-threonine kinase that is mainly used as a metabolic sensor to coordinate anabolic and catabolic processes in the heart (Shirwany and Zou, 2010). Studies have indicated that stimulation of AMPK during myocardial ischemia is useful for cardiomyocytes' survival, which may be due to decreased apoptotic process, elevated ATP production, and enhanced glucose and fatty acid metabolism (Bairwa et al., 2016). Indeed, earlier work indicated that neonatal cardiomyocytes treated with resveratrol to stimulate AMPK became resistant to high glucose-induced apoptosis and that this anti-apoptotic impact weakens when cardiomyocytes are exposed to Compound C to inhibit AMPK (Guo et al., 2015). Consistent with this, continuous isoproterenol stimulation inhibits AMPK phosphorylation, leading to increased apoptosis, and pharmacological stimulation of AMPK via metformin eliminates apoptosis in isoproterenol-induced (Zhuo et al., 2013). Our study showed that lncRNA Chaer significantly enhanced the activity of AMPK in cardiomyocytes treated with OGD, thus reduced cardiomyocyte apoptosis. However, this effect could be reversed by the administration of AMPK inhibitor, Compound C. These results suggested that lncRNA Chaer might reduce cardiomyocytes apoptosis by enhancing the activity of AMPK.

Additionally, mTOR is mainly considered to be downstream of AMPK (Yan et al., 2019), a nutrition and growth factor sensing complex that has a considerable role in cell growth and development (Sciarretta et al., 2018). The reported studies have indicated that oxidative stress activated the mTOR cascade, leading to cell death of cardiomyocytes, while AMPK activation protected against oxidative stress by reducing mTOR-regulated anabolic growth to reduce energy requirements (Zhu et al., 2019). Accordingly, Li FP *et al.* validated that lncRNA Chaer could accelerate cell growth and block the apoptotic process in vascular endothelial cells. While Chaer-PRC2 interactions were found to be transiently induced via stress or hormonal stimulation in an mTOR dependent manner. The blockage of the mTOR signaling cascade could reduce PRC2 activity, thereby promoting cell proliferation and inhibiting apoptosis (Wang et al., 2016). Consistent with the above studies, we found that OGD-treated cardiomyocytes blocked the AMPK phosphorylation and elevated the mTOR phosphorylation, while lncRNA Chaer stimulated AMPK phosphorylation and blocked mTOR phosphorylation to reduce cardiomyocytes apoptosis.

There are still some questions in this study that have not been addressed. First, although the data indicated that lncRNA Chaer protected cardiomyocytes from AMI injury by stimulation of the AMPK/mTOR cascade, whether lncRNA Chaer directly acts on specific subunits of AMPK (Sun et al., 2018) remains to be investigated, and this will be our further studies. Second, studies have shown that Chaer-PRC2 interaction-mediated apoptosis was an mTOR-dependent event, and the activity of mTOR could be inhibited by AMPK, thereby inducing the activation of cell autophagy (Ouyang et al., 2014; Dong et al., 2019). Therefore, we hypothesized that lncRNA Chaer might regulate cardiomyocytes apoptosis by inducing cardiomyocytes autophagy, but the specific mechanism requires further elucidation. The detailed mechanism through which lncRNA Chaer stimulates AMPK to regulate cardiomyocytes apoptosis will be the key objective of our future studies.

In conclusion, our study indicated that the overexpression of lncRNA Chaer showed cardioprotective effects against myocardial AMI injury via positively stimulating the AMPK/mTOR cascade and preventing the cell death of cardiomyocytes. LncRNA Chaer might be a candidate target to overcome and cure myocardial ischemic diseases.

## Data Availability

The raw data supporting the conclusions of this article will be made available by the authors, without undue reservation, to any qualified researcher.
