# Supplementary material for: LncRNA Chaer Prevents Cardiomyocyte Apoptosis From Acute Myocardial Infarction Through AMPK Activation
Source: Front Pharmacol. 2021 Jul 15;12:649398. doi: 10.3389/fphar.2021.649398 (PMC8322763; doi:10.3389/fphar.2021.649398)
Supplement: Supplementary file 1 [file DataSheet1.PDF]

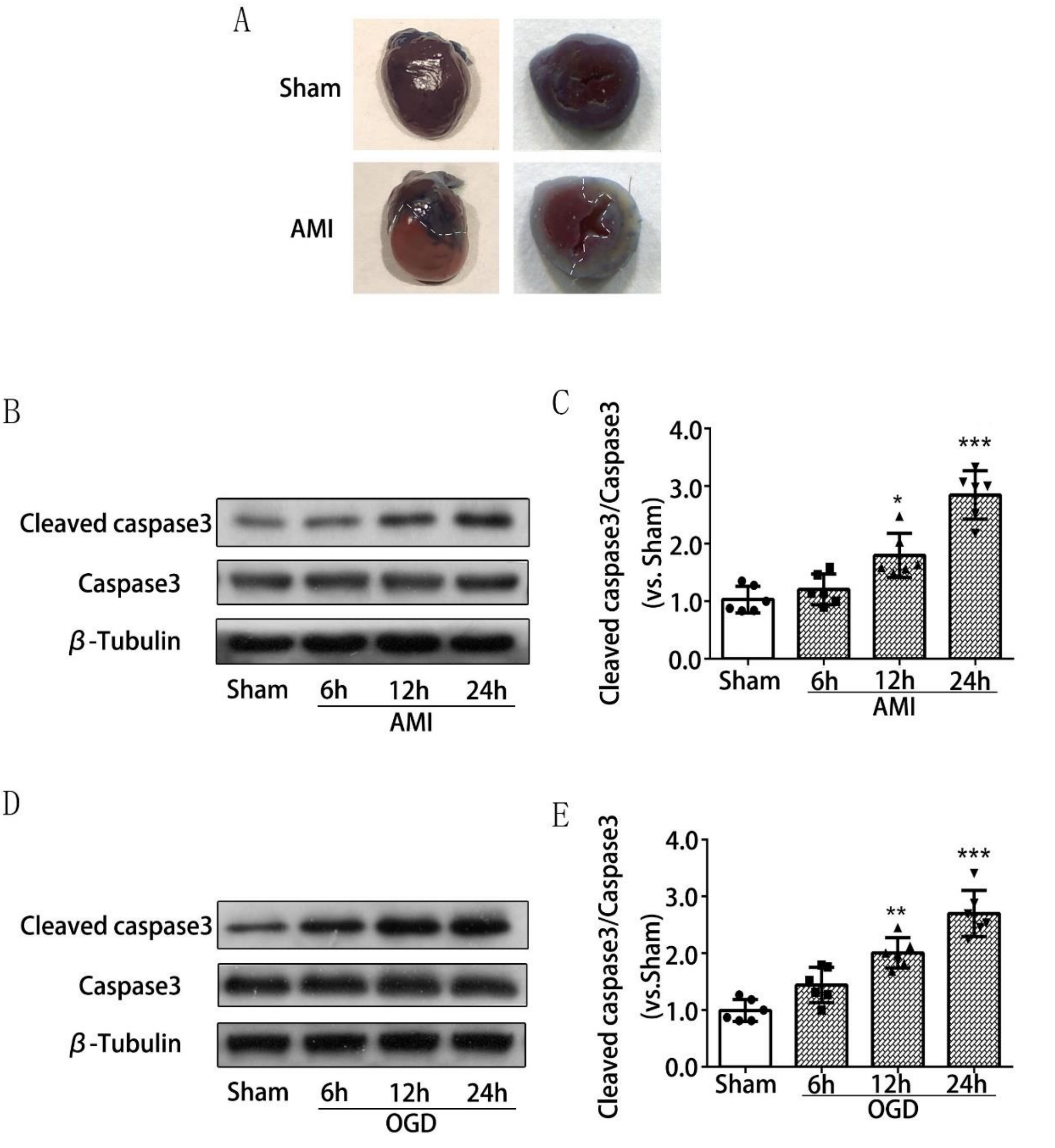

Supplementary figure 1. (A) Representative images of integral heart and middle cross sections of cardiac Evans blue/TTC staining. Blue staining represents the non-infarct area, red staining indicates the area at risk (AAR) and white staining indicates the infarct area (IA). (B-C) Representative Western blotting and quantification of Cleaved caspase3 expression in the infarct border zone of mouse heart post-AMI in a time-dependent manner. **P<0.05, ***P<0.001 vs. sham*, *n=6*. (D-E) Representative Western blotting and quantification of Cleaved caspase3 expression in cardiomyocytes treated with OGD in a time-dependent manner. ***P<0.01, ***P<0.001 vs. sham, n=6.*

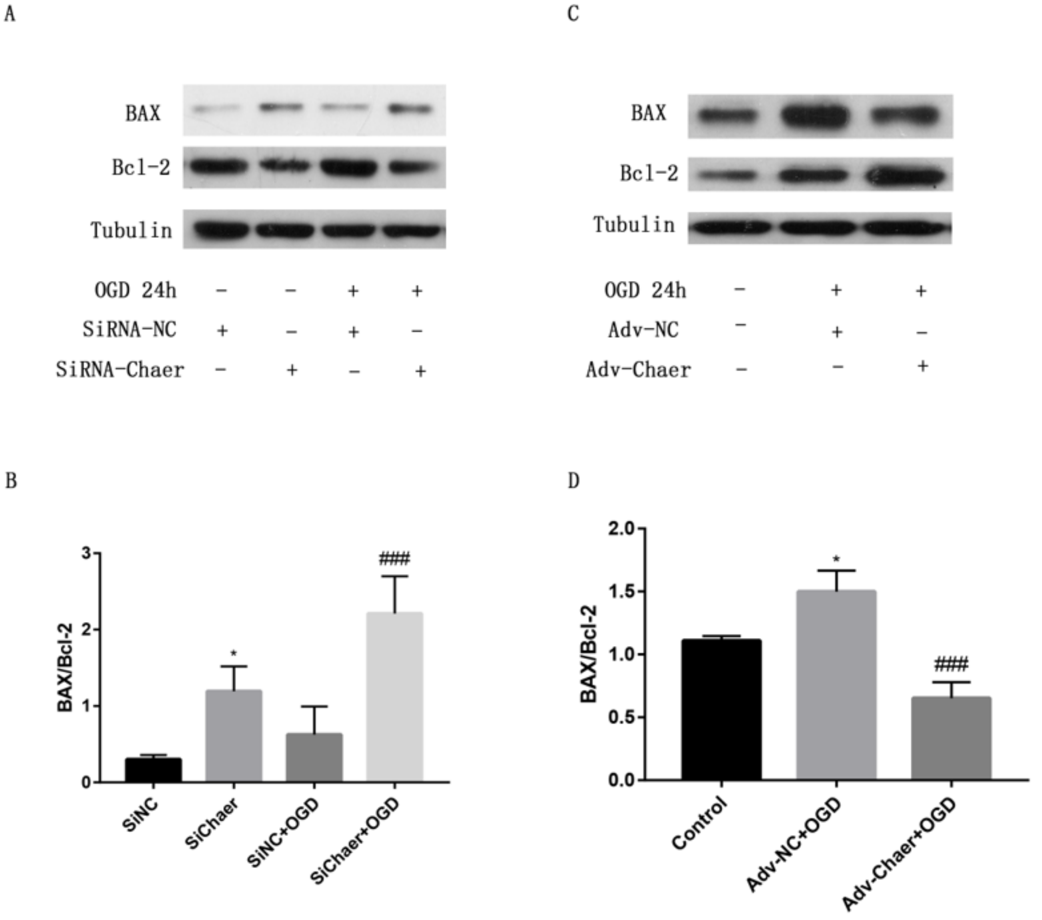

Supplementary figure 2. LncRNA Chaer suppresses the BAX/Bcl-2 ratio in CMs. (A-B) Knockdown of lncRNA Chaer elevated the BAX/Bcl-2 ration in CMs. *P* < 0.05 vs SiNC, P** < 0.01 vs SiNC,* $P^{\#}$ *< 0.05 vs SiNC+OGD,* $P^{\#\#\#}$ *< 0.001 vs SiNC+OGD (n=3)* ; (C-D) Overexpression of lncRNA Chaer decreased the BAX/Bcl-2 ratio in CMs under OGD condition. *P* < 0.05 vs control,* $P^{\#\#\#}$ *< 0.001 vs Adv-NC+OGD (n=3).*
